# Towards understanding the welfare of cetaceans in accredited zoos and aquariums

**DOI:** 10.1371/journal.pone.0255506

**Published:** 2021-08-30

**Authors:** Lisa K. Lauderdale, Jill D. Mellen, Michael T. Walsh, Douglas A. Granger, Lance J. Miller

**Affiliations:** 1 Conservation Science and Animal Welfare Research, Chicago Zoological Society – Brookfield Zoo, Brookfield, Illinois, United States of America; 2 Biology Department, Portland State University, Portland, Oregon, United States of America; 3 Department of Comparative, Diagnostic & Population Medicine, College of Veterinary Medicine, University of Florida, Gainesville, Florida, United States of America; 4 Institute for Interdisciplinary Salivary Bioscience Research, University of California, Irvine, California, United States of America; Universita di Bologna, ITALY

## Abstract

Cetaceans are long-lived, social species that are valued as ambassadors inspiring the public to engage in conservation action. Under professional care, they are critical partners with the scientific community to understanding the biology, behavior, physiology, health, and welfare requirements of this taxonomic group. The Cetacean Welfare Study was a highly collaborative research effort among zoos and aquariums accredited by the Alliance for Marine Mammal Parks and Aquariums and/or the Association of Zoos & Aquariums that provided important empirical and comparative information on the care and management of cetaceans. The goal was to identify factors that were related to the welfare of bottlenose dolphins and to develop reference intervals and values for common and novel indicators of health and welfare for common bottlenose dolphins (*Tursiops truncatus*), Indo-Pacific bottlenose dolphins (*Tursiops aduncus*), beluga whales (*Delphinapterus leucas*), and Pacific white-sided dolphins (*Lagenorhynchus obliquidens*). Data were collected from cetaceans at 43 accredited zoos and aquariums in seven countries in 2018 and 2019. This overview presents a summary of findings from the initial research articles that resulted from the study titled “Towards understanding the welfare of cetaceans in zoos and aquariums.” With multiple related objectives, animal-based metrics were used to advance frameworks of clinical care and target key conditions that were associated with good welfare of cetaceans in zoo and aquarium environments. As a result of this collaboration, species-specific reference intervals and values for blood variables and fecal hormone metabolites were developed and are freely available in an iOS application called ZooPhysioTrak. The results suggested that environmental enrichment programs and social management factors were more strongly related to behaviors likely indicative of positive welfare than habitat characteristics for common and Indo-Pacific bottlenose dolphins. These findings can be widely applied to optimize care and future science-based welfare practice.

## Introduction

Modern accredited zoological facilities manage valuable animal conservation, education, and research programs [1, 2]. Accredited zoos and aquariums prioritize animal welfare in management strategies and research plans [3–5]. Welfare refers to an animal’s collective physical, mental, and emotional states (i.e., holistic health) over a period of time, and is generally measured on a continuum from good to poor [6]. Accredited zoos and aquariums aim to optimize welfare by using scientific research to inform evidence-based decision making [3–5]. Recent peer-reviewed scientific studies detail advancements in validating positive and negative indicators of welfare and developing monitoring tools, particularly in relation to high-profile species such as elephants, primates (i.e., monkeys and apes), and cetaceans (i.e., whales, dolphins, and porpoises) [7–10]. In addition, multi-institutional research has become critical to investigating animal welfare due to the diverse habitats and management practices used to care for these species. Notably, one recent multi-institutional study was published as a collection of papers titled *Epidemiological Investigations of North American Zoo Elephant Welfare* and examined elephant welfare in 68 zoos. This collection provided a framework for the holistic study of welfare across a large number of zoos [11] and served as a base model for research on cetacean welfare due to the similarities in managing and studying large, cognitively complex species.

Professionally managed zoo/aquarium and ocean habitats differ in size, shape, water source, and natural or fabricated environmental features [12]. Common bottlenose dolphins (*Tursiops truncatus*), Indo-Pacific bottlenose dolphins (*Tursiops aduncus*), beluga whales (*Delphinapterus leucas*), and Pacific white-sided dolphins (*Lagenorhynchus obliquidens*) are some of the most common cetacean species under professional care [12, 13]. Zoos and aquariums housing cetaceans are regulated by their respective governments; some are also accredited by professional organizations such as the Alliance for Marine Mammal Parks and Aquariums (AMMPA) and the Association of Zoos & Aquariums (AZA). Cetacean-holding zoos and aquariums accredited by the AMMPA and AZA meet high standards of care and implement programs that specifically evaluate and manage the welfare of the animals in their care. Accreditation standards for the AMMPA and AZA require that zoos and aquariums have education programs based on current scientific knowledge, follow enrichment programs that promote species-appropriate behavioral opportunities, and implement formal positive reinforcement-based training programs that are developed to enhance the health and welfare of the animals.

Interest in research on cetacean welfare has focused on identifying husbandry and veterinary practices that optimize welfare. Contemporary cetacean welfare science incorporates both physiological and behavioral measures. Researchers have examined the relationship between a range of input and output variables that may be associated with welfare. Input variables have included, but are not limited to, habitat size [14, 15], habitat access [16], training [4, 17, 18], and environmental enrichment [19–22]. Outcome variables that may be indicators of welfare have included, but are not limited to, behavior [23], morphometrics [24], glucocorticoid levels [25], and blood panels [26]. This extensive list has supplied researchers with a foundation for examining cetacean welfare on a large scale.

There is a strong commitment among zoos and aquariums to continuously advance an understanding of welfare across facilities using scientific methods to positively impact the quality of life for the animals. Zoos and aquariums require objective information on the state of cetacean welfare that is representative of current management practices and the animals’ diverse habitat types to move welfare forward. The overarching goals of the research presented within this collection was to identify factors that were associated with positive welfare, build tools to track and monitor welfare long-term, and provide scientific, welfare-relevant data that lays the foundation for the continuous improvement of care and future research.

## Cetacean welfare study: A multi-institutional investigation of the welfare of cetaceans in accredited zoos and aquariums

This collection of papers represents the data generated from the Cetacean Welfare Study, which was the largest multi-institutional study examining the welfare of cetaceans in accredited zoos and aquariums to date. This inter-disciplinary assessment of welfare for common bottlenose dolphins, Indo-Pacific bottlenose dolphins, beluga whales, and Pacific white-sided dolphins included zoos and aquariums on four continents. The first goal of the Cetacean Welfare Study was to collect data to develop species-specific reference intervals and values for traditional and novel physiological biomarkers of health and welfare by compiling physiological health data from the four study species. The second goal was to identify welfare-related factors of bottlenose dolphins to understand their association with physical habitat, environmental enrichment, and animal training. Data were collected on the husbandry practices and housing conditions at 43 zoos and aquariums accredited by the AMMPA and/or AZA.

**Fig 1:**
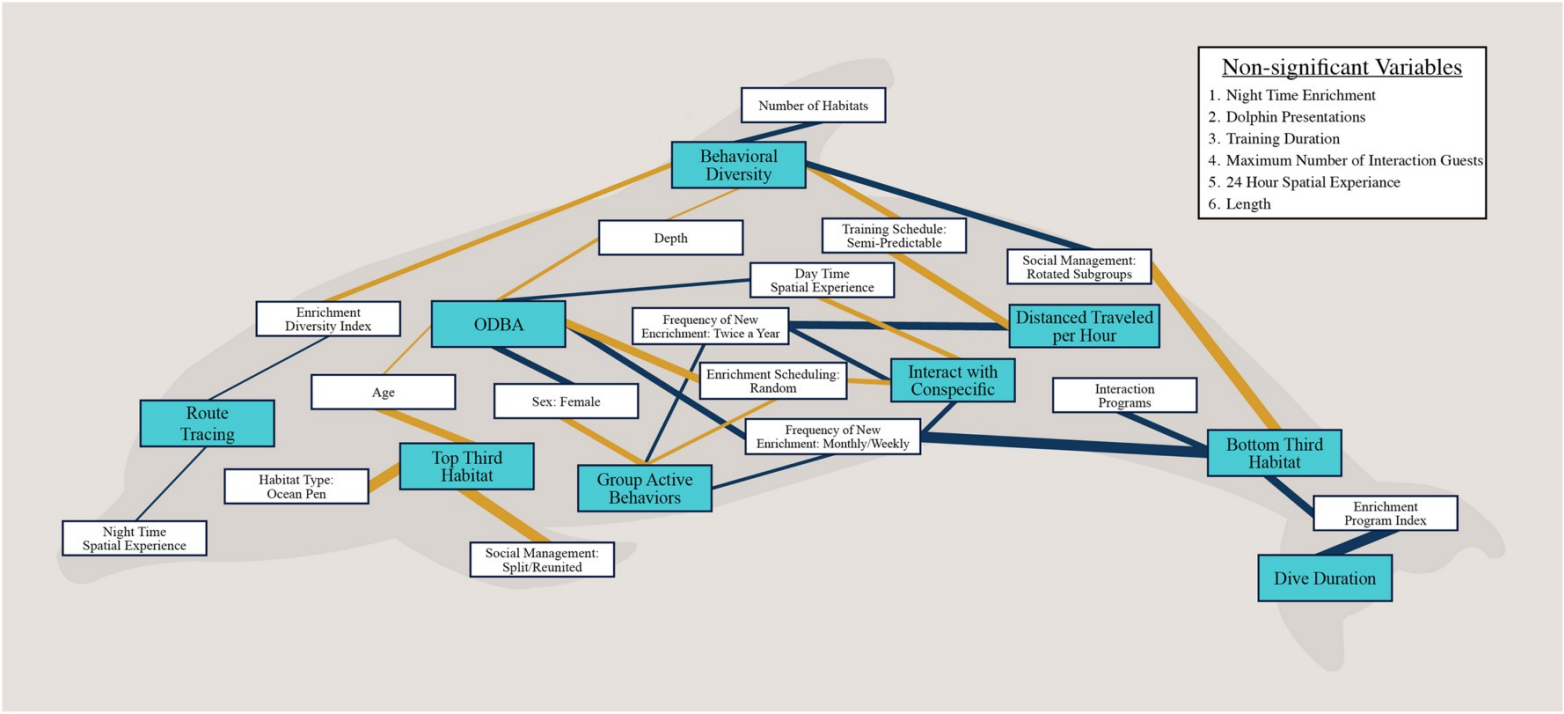
Welfare outcome and independent variable associations. Outcome variables are shown in blue boxes and independent variables are shown in white boxes (variables are defined in [27]). Positive correlations are represented with blue connecting lines and negative correlations are represented with yellow connecting lines. The strength of the relationship is indicated by the thickness of the connecting line.

**Fig 2:**
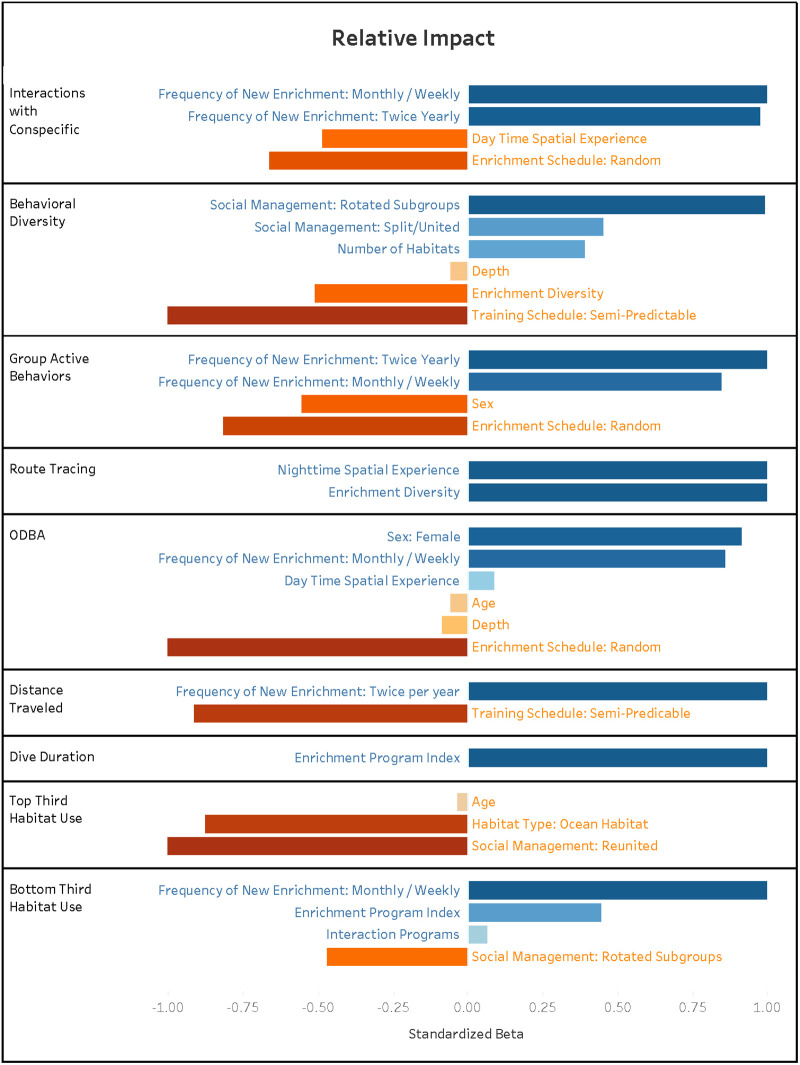
Relative impact outcome and independent variable associations for all significant variables within the collection. Beta values were standardized by dividing the Beta value by the maximum Beta value of each individual model. Directional effects are represented as positive and negative associations with the dependent variable. Positive associations indicate an increase in the dependent variable and negative associations indicate a decrease in the dependent variable.

An overview of significant relationships for welfare outcomes and independent variable associations for all research articles is provided in Fig 1. Figures highlighting relationships between input variables (i.e., demographic, environmental enrichment, training, and habitat characteristic variables) and behavioral outcome variables is provided in S1 File. Details on the directionality and relative impact of significant relationships are provided in Fig 2. Major study findings are discussed below, and avenues of future research are suggested.

### Goal 1: Develop health reference intervals and values

All accredited zoos and aquariums that care for cetaceans have developed health monitoring and preventative care programs that include monitoring multiple aspects of physical health [24–26, 28–30]. Diagnostic testing is conducted on biological samples collected during routine examinations to assess health and monitor welfare. For example, comprehensive blood tests are useful for detecting physical health abnormalities. Veterinarians compare an individual’s results to previously established clinicopathologic reference intervals for that individual and to other healthy individuals of that species [26, 30].

Cetaceans are commonly trained to provide voluntary blood samples that can be used to inform physical health status and treatment plans. Blood values for numerous health indicators in common bottlenose dolphins and beluga whales have been associated with age, sex, and season [26, 30]. Lauderdale et al. [31] presents reference intervals and values from a sample of healthy common bottlenose dolphins, Indo-Pacific bottlenose dolphins, beluga whales, and Pacific white-sided dolphins. Biological samples were analyzed by one of two laboratories. Cetaceans were classified as healthy by expert veterinarians and pathologists based on blinded results from full physical examinations and cytology findings. These hematological, serum, and plasma reference intervals and values provide an important comparative diagnostic reference tool set for future health assessments for cetaceans in zoos and aquariums. Sex, age, age^2^, and month predictor variables were found to be important determinants of several blood variables and understanding these variables is important in making proper medical decisions on intervention and therapy.

Previous research investigating cetacean welfare has largely focused on blood values [32–34]. However, examining fecal hormone metabolite values provides an important method of measuring and monitoring adrenal hormones on a long-term basis. Fecal samples are collected non-invasively which allows samples to be obtained more often than blood samples. Specifically, tracking an individual’s fecal hormone levels can be used to establish baseline hormone levels and referenced to evaluate adrenal response to environmental or social changes [35]. Regular tracking of fecal hormone metabolites also allows animal care specialists to monitor long-term adrenocorticoid activity by using animals as their own controls in a variety of situations.

There are several corticoid biomarkers from feces, including cortisol, aldosterone, and dehydroepiandrosterone (DHEA), that may be associated with animal welfare. Cortisol, a glucocorticoid produced by the adrenal glands, supports the body’s response to stress, exercise, and arousal [36]. Aldosterone is a mineralocorticoid produced by the adrenal glands that works as part of the hypothalamic-pituitary-adrenal axis response to stabilize blood pressure [37]. DHEA is a glucocorticoid antagonist whose levels have been linked to knowledge retention, anti-aging properties, immune system enhancement, and mental health in humans [38–40]. Fecal corticoid metabolites for aldosterone and DHEA are markers of adrenal activity and, when examined collectively with cortisol, are potentially relevant in monitoring an individual’s adrenal function over time. While these markers show promise as potential indicators of welfare, they have not been validated in cetaceans and data from the Cetacean Welfare Study serves as a starting point to provide baseline values.

For the Cetacean Welfare Study, expert veterinarians and pathologists reviewed blinded results from two full physical examinations, complete blood counts, serum chemistry, and cytology findings to gauge each animal’s health status. Sex, age, and/or the month the sample was collected (i.e., seasonality) were found to be predictors of fecal cortisol, aldosterone, or DHEA metabolites in several of the regression models. However, sex, age, and month explained very little of the variance in the regression models indicating that other factors may have a stronger effect on hormone concentrations. Trait (i.e., commonality of an individual’s biomarker levels over time) and state (i.e., time-specific variations in an individual’s biomarker levels) related predictors of hormone metabolic values may provide more insight into the variance seen in the data. In humans, elevated serum cortisol:DHEA ratios have been attributed more strongly to trait-anxiety than to clinical symptoms suggesting that the hypothalamic-pituitary-adrenal axis is independent of clinical state [41]. Fecal hormone metabolite reference intervals and values for healthy common bottlenose dolphins, Indo-Pacific bottlenose dolphins, beluga whales, and Pacific white-sided dolphins are presented in Miller et al. [42].

Easy access to information about comparative health parameters for each species is critical for zoo and aquarium staff to utilize scientific data when making health care decisions. Therefore, ZooPhysioTrak, an iOS mobile software application, was developed to distribute the species-specific reference intervals and values alongside the ranges published in Lauderdale et al [31] and Miller et al. [42]. ZooPhysioTrak allows users to input individual demographic information about any individual and view reference intervals or values generated from the blood and fecal samples that are fitted to a specific demographic profile. Future studies should continue to bolster the number of samples included in the reference values, particularly for beluga whales and Pacific white-sided dolphins, and investigate the impact of other potential predictors such as diet.

### Goal 2: Investigate the relationship between habitat characteristics, management practices, and welfare

In recent decades, animal welfare science has evolved rapidly and has been used to advance evidence-based management in zoos and aquariums [3–5]. Facilities accredited by the AMMPA and AZA maintain high standards of care and implement programs that specifically evaluate and manage animal welfare [2]. Due to the variability in habitat types and management practices, a multi-institutional approach is key to understanding bottlenose dolphin welfare; large sample sizes provide a pathway to developing a holistic understanding of correlates to good welfare.

Lauderdale et al. [27] provides a review of the current habitats and management practices at the zoos and aquariums that participated in the Cetacean Welfare Study. Animal care specialists at participating facilities completed a management survey. Differences in habitat characteristics and management practices between facilities were identified and quantified. Information from the management survey was used to create several direct and synthesized variables that characterized demographic features, habitat features, environmental enrichment programs, and training programs for bottlenose dolphins that are potentially associated with welfare based on prior research [3, 4, 8, 11]. These input variables (i.e., demographic, environmental enrichment, training, and habitat characteristic variables) were then used to examine associations with behavioral outcomes (i.e., various measures of activity, behavior, and habitat use) in Lauderdale et al. [43, 44] and Miller et al. [45–47]. The demographic variables that were examined included the sex and age of the dolphins. The environmental enrichment program variables that were examined included: Enrichment Diversity Index, Enrichment Program Index, Night Time Enrichment, Enrichment Schedule, and Frequency of New Enrichment. The training program variables that were examined included: Dolphin Presentations, Interaction Programs, Training Duration, Maximum Number of Interaction Guests, and Training Schedule. The habitat characteristics that were examined included: Day Time Spatial Experience, Night Time Spatial Experience, 24 Hour Spatial Experience, Length, Depth, Habitat Type, Number of Habitats, Social Management, and Neighboring Conspecifics. See S1 Table for definitions of the independent variables used in the analyses.

#### Demographics

Bottlenose dolphins are behaviorally complex, social species that live in fission-fusion societies [48, 49]. Demographic characteristics such as the age and sex of an individual may impact its social life and activities [50–52]. In wild dolphin populations in Florida and Australia, females typically socialize with other females in their groups while male bottlenose dolphins often form strong bonds with other males to form an alliance [50–53]. Age may also influence an individual’s activities. For example, younger dolphins under professional care engage in more play behavior while older animals engage in more low intensity swimming [54].

For dolphins participating in the Cetacean Welfare Study, overall dynamic body acceleration (ODBA; a proxy for energy expenditure) decreased with age. The decreasing ODBA values of older dolphins is consistent with previous reports that high energy activities such as play are more common in younger dolphins [44]. Older dolphins also used the top third of the habitat less than younger animals which suggests that dolphins may use the bottom of the habitat for low intensity solitary or group swimming [43]. One explanation may be that older animals learn to reduce their activity levels by diving to deeper depths where drag is reduced [55]. Similarly, previous reports found that dolphins heavily favored swimming in the bottom two thirds of the habitat when engaging in slow, resting swimming patterns [56]. In addition, females had significantly higher ODBA values, yet engaged in less group active behaviors than males across facilities [44, 46]. This finding has not been previously reported and both females and males are generally considered to be highly social [57, 58].

#### Environmental enrichment

Much of the previous research that has been conducted in zoos and aquariums has focused on examining the efficacy of environmental enrichment. Environmental enrichment is defined as the addition of objects, stimuli, or activities into an animal’s environment with the goal of eliciting species-appropriate behaviors and presenting opportunities for choice and control [59–61]. Zoos and aquariums employ environmental enrichment programs to improve animal welfare by promoting species-appropriate behavioral opportunities [19, 62–64].

Cetacean environmental enrichment programs are comprised of a wide collection of goal-related activities such as the addition of floating or sinking objects to a habitat to encourage investigation and swimming (e.g., television, balls, and underwater mazes) [19, 20, 65], training programs for mental stimulation [66], and social changes aimed at improving welfare [67]. Programs can also utilize cognitively challenging enrichment to delay habituation, reinforce and stimulate problem solving, and expand our understanding of cognition [62, 68]. Challenging tasks are effective forms of enrichment that can increase social behaviors and the amount of time spent underwater [19]. Enrichment that can be used by multiple dolphins simultaneously has also been shown to increase the time spent at the bottom of the habitat and increased social swimming even when the enrichment was not present [22]. To ensure that environmental enrichment is effective at achieving the intended outcome, the program and its components must be regularly evaluated. One method used by zoos and aquariums to develop and evaluate an environmental enrichment program (as opposed to assessing an individual type of enrichment) is through the application of a SPIDER model [69]. A SPIDER model is a framework for developing successful enrichment programs that includes six steps: Setting Goals, Planning, Implementing, Documenting, Evaluating, and Readjusting.

For the Cetacean Welfare Study, environmental enrichment variables accounted for 14 of the 31 significant relationships that were observed [43–46]. Environmental enrichment variables were significantly related to all the behavioral outcomes examined except for use of the top third of the habitat. Of these variables, how often dolphins received new enrichment was associated with group active behaviors, interactions with conspecifics, ODBA, distance traveled, and use of the bottom third of the habitat [43, 44, 46]. This variable may have been particularly important as receiving new enrichment on a monthly/weekly or biannual schedule was associated with increases in measures of energy expenditure, positive social relationships, and use of the bottom third of the habitat. The schedule in which the enrichment was provided was also related to similar variables. Dolphins provided enrichment on a predictable schedule had higher ODBA values, engaged in group active behaviors more often, and interacted with conspecifics more often than those provided enrichment on a non-predictable schedule [44, 46].

Markedly, the enrichment program index values were only related to habitat use variables. The enrichment program index was a synthesized score created based on the frequency with which facilities engaged in several evaluative aspects of their enrichment programs. The features of environmental enrichment programs (e.g., setting goals and evaluating enrichment) were not only related to increased activity levels, but also were related to longer diving bouts. This may be due in part to the effort to develop sinking environmental enrichment that are designed to stimulate use of the full depth of the habitat [22].

The enrichment diversity index was not associated with any of the activity or habitat use variables that were related to the other environmental enrichment variables. The enrichment diversity index was a value that represented how diverse an enrichment program was while accounting for the number of different types of enrichment as well as how often each type was provided. Neither of the findings that higher enrichment diversity index values were associated with increased route tracing and decreased behavioral diversity would be expected based on previous research on environmental enrichment [70, 71]. However, an inverse relationship has been previously observed between route tracing and behavioral diversity in bottlenose dolphins [47]. Given that route tracing was infrequently observed and not related to any other environmental enrichment variable, it is possible that this was a result of the animal care staff providing more diverse enrichment in an attempt to decrease a pre-existing behavior as opposed to enrichment eliciting the behavior. Providing environmental enrichment has previously been shown to successfully reduce stereotypic behavior [72–75]. Therefore, it is feasible that facilities were implementing this strategy by providing an array of enrichment to decrease route tracing and increase behavioral diversity.

Behavioral diversity refers to the frequency and richness of an individual animal’s behavior [28]. Prior research has laid the groundwork for the potential of behavioral diversity to be used as an indicator of welfare [76–79]. While behavioral diversity has yet to be validated as an indicator of positive welfare for any species, the underlying hypothesis is that if the animal’s needs are satisfied, it would exhibit high behavioral diversity (i.e., frequently exhibits a variety of species-appropriate behaviors). Alternatively, restricting behavioral opportunities would presumably result in low behavioral diversity (i.e., the animal would be exhibiting few behaviors) as stereotyping or lethargy are typical signs of poor welfare [80, 81]. Terrestrial mammals such as horses, pigs, mink, and mice display low behavioral diversity when their behavior has been restricted compromising their welfare [29, 82–84]. Thus, behavioral diversity is an important variable to consider in cetacean welfare research.

#### Training

Training programs at accredited facilities are based on current methods and best practices for the application of positive behavioral and training (learning principle) welfare programs. Cetaceans under professional care are often trained to participate in their own health care, educational presentations, and beneficial research studies by learning a variety of behaviors. Whales and dolphins have been trained for a wide array of voluntary husbandry/medical behaviors including allowing full body examinations, ultra-sounds, dental cleaning, blood sampling, urine sampling, gastric sampling, fecal sampling, and respiratory sampling (see [17] for a comprehensive review of husbandry training in marine mammal programs). Training these behaviors to provide preventive healthcare and treatment procedures decreases the need for physical restraint that could potentially be associated with negative experiences and impact welfare without medical training. For example, cortisol levels were significantly reduced when harbor porpoises (*Phocoena phocoena*) were trained to voluntarily participate in the collection of biological samples when compared to samples collected when the animals were removed from the water for collection [85]. Therefore, teaching husbandry behaviors using positive reinforcement is now integral to training programs [17].

Many zoos and aquariums with cetaceans offer interactive experiences such as educational presentations, swim with dolphin/whale programs, or dockside interactive programs. Whales and dolphins can be taught specific behaviors that allow visitors to be in close proximity and learn more about these species during educational presentations [86, 87]. The interactive programs and public presentations may be beneficial to dolphin welfare as well. In a study of bottlenose dolphins at six zoos, Miller and colleagues [4] found that dolphins exhibited higher rates of behavioral diversity, diversity of swimming style, and play behavior following presentations and interaction programs. Affiliative behavior, aggressive behavior, and repetitive behavior were unrelated to public presentations or interaction programs. Other similar studies have found increases in play behaviors after interaction programs and have described no adverse behavior changes [88, 89].

In the Cetacean Welfare Study, results showed that training variables were related to behavioral diversity, distance traveled per hour, and use of the bottom third of the habitat [43–45]. Dolphins trained on a predictable schedule had higher behavioral diversity and higher distance traveled per hour when compared to those trained on a semi-predictable schedule [44, 45]. Dolphins who participated in a larger number of interaction programs used the bottom third of the habitat more often [43]. However, the magnitude of change in bottom third habitat use per interaction program was small. As most facilities with professionally managed ocean habitats offered interaction programs rather than presentations, it is possible that this is an artifact of professionally managed ocean habitats being an average of 3.15 m shallower than professionally managed zoo/aquarium habitats in the study.

#### Habitat characteristics

Scientific examination of how animals interact with their habitat can be used to determine habitat appropriateness [90, 91]. Habitats that are an appropriate size, have multiple accessible areas, and include environmental enrichment have been associated with positive welfare [92–94]. While increased locomotion and greater distance traveled for several terrestrial species has been positively correlated with larger enclosure sizes [95], continued expansion of habitat size for some species does not always correspond to increased locomotion [96]. Bottlenose dolphins in accredited zoos and aquariums typically experience a range of habitat types and management programs that are designed to provide opportunities for dolphins to engage in species-appropriate behaviors. This study investigated correlates between welfare measures and size/complexity/access to dolphins’ habitats.

In zoos and aquariums, length and depth of habitats have been suggested to impact behavior. When one area of the habitat is available at a time, relatively larger habitats have been associated with reduced aggression and higher swimming rates [14, 97, 98]. When areas with varying depths were available, bottlenose dolphins chose to use the moderate and shallow depth areas 98% of the time [15]. The horizontal dimension of the habitat has been shown to be more related to positive (i.e., non-stereotypic) behaviors than other habitat dimensions [14].

In the Cetacean Welfare Study, habitat characteristics were related to social behaviors, habitat use, and stereotypic behaviors [43–46]. Dolphins with larger daytime spatial experience values (i.e., the proportionate volume of water available during the day) had fewer interactions with conspecifics and had higher ODBA values [44]. However, these associations were very weak based on the beta coefficients when compared to sex and enrichment variables. ODBA was also related to the maximum depth of the habitat. Deeper maximum depths in habitats were associated with lower ODBA values and lower behavioral diversity values. Deeper depths may have been associated with lower ODBA values because less active swimming is required while swimming at the bottom of the habitat and during the ascent portion of a dive sequence than what is required for multiple short surfacing events [99].

Dolphins with access to a larger number of habitats during the cetacean welfare study had higher behavioral diversity values [45]. Research with other species has shown that having the ability to distance self both physically and visually from other animals, can lead to positive welfare [100, 101]. Bottlenose dolphins have a recognized dominance hierarchy [87] and, therefore, may have benefitted from the ability to physically distance themselves from other group members or from having multiple areas from which to choose where to spend their time. Dolphins that were managed in groups that were rotated through subgroups also had higher behavioral diversity and used the bottom third of the habitat less often when compared to dolphins managed in the same group. Dolphins that were managed in groups that were split and reunited used the top third of the habitat less often than those in the same group all the time. Dolphins managed in using the split/reunited and rotated subgroup methods may more closely mimic their natural history as wild bottlenose dolphins are a complex social species that live in a fission-fusion society [102].

Dolphins in professionally managed ocean habitats swam in the top third of the habitat less often than dolphins in professionally managed zoo/aquarium habitats which may be a result of a number of factors [43]. This may have been due to animals exploring natural flora and fauna in the ocean environment. It is also feasible that this may be attributed to the mean maximum depth of the participating professionally managed zoo/aquarium habitats being deeper than professionally managed ocean habitats resulting in each third of the habitat including a smaller relative distance. In addition, time spent in the top third of the habitat may have been moderated by access to floating and sinking enrichment and/or by the fact that dolphins received food from animal care staff at the surface. Another viable explanation may be related the proximity of animal care staff and/or guests throughout the day at various habitats. While future research should continue to investigate this topic, use of the top third of the habitat was the only behavioral outcome variable that was investigated that was related to habitat type. This suggested that dolphin’s overarching activity levels, social behavior, and habitat use were not greatly modified by residing in professionally managed ocean habitats when compared to professionally managed zoo/aquarium habitats.

## Strengths and limitations

These articles contribute to our understanding of cetacean welfare and provide a foundation for future investigations by identifying potential relationships between welfare and environmental enrichment, training, and management practices. The findings are strengthened by the large, representative cohort of accredited facilities and individual participants. The use of non-invasive bio-loggers (MTags) provided another avenue of research by enabling the quantification of activity and movement on an incredibly fine scale which could not be acquired by observers alone. In addition, the sample size and standardized collection and processing of biological samples facilitated the development of the ZooPhysioTrak iOS application. ZooPhysioTrak provides blood and fecal hormone metabolite reference intervals and values that can be consulted by veterinarians when assessing results of future tested samples, compared to in-house reference values, used to guide health assessments, and encourage innovation.

Although this collection contributes significantly to the field of cetacean welfare science, the work related to dolphin behavior that is presented is limited by the correlational design. The present study utilized a non-experimental approach due to the variability in management (e.g., environmental enrichment and training programs) and habitat characteristics among the many accredited facilities. This allowed for the exploration of multiple factors that may be associated with welfare. Findings reported from the articles in this collection should serve as a foundation for future research, which could clarify the cause and direction of these associations.

## Impact

The goal of zoo animal welfare science is to continuously improve knowledge and tools related to the assessment of health and welfare of species under professional care. Evidence-based practice is the explicit use of the most current objective information on a topic to make appropriate science-based decisions. Using this decision-making framework means integrating scientific evidence with the finest available diagnostic tools and clinical/husbandry expertise to produce the best outcomes for animals under professional care. Evidence-based management of animals in zoos and aquariums is critical in providing the best possible welfare. Zoo management professionals continually work to fill the gaps in knowledge and enhance best practice. The Cetacean Welfare Study exemplifies the cetacean community uniting in this goal. The present research collection aids in presenting options for improved management decisions, informing future welfare practice, providing tools to assist in making health care decisions, and directing future research between indicators of welfare and the daily lives of cetaceans. This forward-thinking management framework can also be used by government personnel and agencies to work with husbandry and veterinary medicine experts to inform regulation based on scientific evidence to meet the highest welfare standards.

## Future directions

This collection of papers represents the first large-scale study to examine the current state of cetacean management and welfare in accredited zoos and aquariums and provide the foundation for future understanding and techniques for additional improvement. While there have been many advancements in marine mammal welfare science over the years, this research reflects a multidisciplinary, multi-institutional investigation of factors associated with welfare that encompass the breadth of habitat types and management practices in accredited zoos and aquariums. In addition, physiological reference intervals and values for healthy common bottlenose dolphins, Indo-Pacific bottlenose dolphins, beluga whales, and Pacific white-sided dolphins are established. These metrics, reference intervals, and reference values can inform future evidence-based management, health care, and habitat design across species and locations.

The goal of this work was to illuminate relevant topics for continued development of a holistic understanding of welfare. To further advance cetacean health research, topics for future studies include identifying novel biological indicators of welfare, improving the quantification of activity, and refining the relationship between different types of social behavior and welfare outcomes. In addition, future research could employ intra-individual repeated assessments to enable the separation of trait versus state components of the variance in physiological biomarkers. Ultimately, we hope this research enhances evidence-based care, drives the future use of new technologies in identifying factors that lead to optimal welfare, and promotes forward progress in validating new measures of welfare (e.g., behavioral diversity). Future studies will refine the associations identified, expand their welfare applications using experimental methods, and incorporate other measures of welfare.

## Supporting information

S1 Table Independent variables lauderdale overview. Definitions of independent variables included in the analyses.
(DOCX)

S1 File Independent variable relationships lauderdale overview. Interactive pdf file highlighting the relationships between demographic, environmental enrichment, training, and habitat characteristic variables.
(PDF)

S1 Fig Striking image lauderdale overview. (TIFF)
