# Supplementary material for: Towards understanding the welfare of cetaceans in accredited zoos and aquariums
Source: PLoS One. 2021 Aug 30;16(8):e0255506. doi: 10.1371/journal.pone.0255506 (PMC8404978; doi:10.1371/journal.pone.0255506)
Supplement: S1 Table — Definitions of independent variables included in the analyses. (DOCX) [file pone.0255506.s001.docx]

S1 Table. Independent variables included in the analysis.

| Variable | Definition | Values | Type of Variable |
| --- | --- | --- | --- |
| ***Demographic*** |  |  |  |
| Sex | Sex of the dolphin | Male/Female | Factor |
| Age | Age of the dolphin | Years | Covariate |
| ***Environmental Enrichment*** |  |  |  |
| Enrichment Diversity Index | Enrichment diversity index was created using the Shannon diversity index on the mean number of days each enrichment is provided at the facility | Index | Covariate |
| Enrichment Program Index | Enrichment program index is a standardized factor score created with scores on frequency of enrichment program components used at the facility using a polychoric PCA | Index | Covariate |
| Night Time Enrichment | Mean number of nights in a week that enrichment was provided to the dolphins at the facility | Number of Nights | Covariate |
| Enrichment Schedule | Categorical value indicating how enrichment was scheduled at the facility | Predictable, Semi-Random, Random | Factor |
| Frequency of New Enrichment | Categorical frequency that a facility provided the dolphins with new types/forms of enrichment | Weekly/Monthly, Twice a Year, Yearly/Year+ | Factor |
| ***Training*** |  |  |  |
| Dolphin Presentations | Mean number of dolphin presentations an individual dolphin participated in each week | Mean Number of Presentations | Covariate |
| Interaction Programs | Mean number of dolphin interaction programs an individual dolphin participated in each week | Mean Number of Interactions | Covariate |
| Training Duration | Mean amount of time each dolphin interacted with an animal care professional for presentations, interaction programs, training sessions, research, or other training activities each week | Hours | Covariate |
| Maximum Number of Interaction Guests | Maximum number of participants allowed for an interaction program for that facility | Number of Participants | Covariate |
| Training Schedule | Categorical variable indicating if the training schedule for the dolphins at that facility was predictable or semi-predictable | Predictable, Semi-Predictable | Factor |
| ***Habitat Characteristics*** |  |  |  |
| Day Time Spatial Experience | Proportionate volume of water the dolphin had access to based on the percentage of daytime hours spent in different habitats in each five-week data collection period | Megaliter | Covariate |
| Night Time Spatial Experience | Proportionate volume of water the dolphin had access to based on the percentage of night time hours spent in different habitats in each five-week data collection period | Megaliter | Covariate |
| 24 Hour Spatial Experience | Proportionate volume of water the dolphin had access to based on the percentage of hours throughout the entire day spent in different habitats in each five-week data collection period | Megaliter | Covariate |
| Length | The maximum straight length in any direction across any habitat the dolphin had access to in each five-week data collection period | m | Covariate |
| Depth | The maximum depth for any habitat the dolphin had access to in each five-week data collection period | m | Covariate |
| Habitat Type | Categorical variable indicating the dolphin was in a professionally managed zoo/aquarium habitat or a professionally managed ocean habitat | Zoo/Aquarium, Ocean | Factor |
| Number of Habitats | Maximum number of habitats (different enclosures) dolphin had access to in daytime hours during each five-week data collection period | Number of Habitats | Covariate |
| Social Management | Categorical variable indicating the type of social management practice for a dolphin during each five-week data collection period | Same Group, Split/Reunited, Rotated Subgroups | Factor |
| Neighboring Conspecifics | Categorical variable indicating if the dolphin had visual and auditory access to other dolphins without possibility of physical contact during each five-week data collection period | No, Yes | Factor |
