# Supplementary material for: Towards understanding the welfare of cetaceans in accredited zoos and aquariums
Source: PLoS One. 2021 Aug 30;16(8):e0255506. doi: 10.1371/journal.pone.0255506 (PMC8404978; doi:10.1371/journal.pone.0255506)
Supplement: S1 File — Interactive pdf file highlighting the relationships between demographic, environmental enrichment, training, and habitat characteristic variables. (PDF) [file pone.0255506.s002.pdf]

Cetacean Welfare Study

Independent Variable Relationships

Click to see the significant variables.

All Variables | Demographic | Environmental Enrichment | Training | Habitat Characteristics

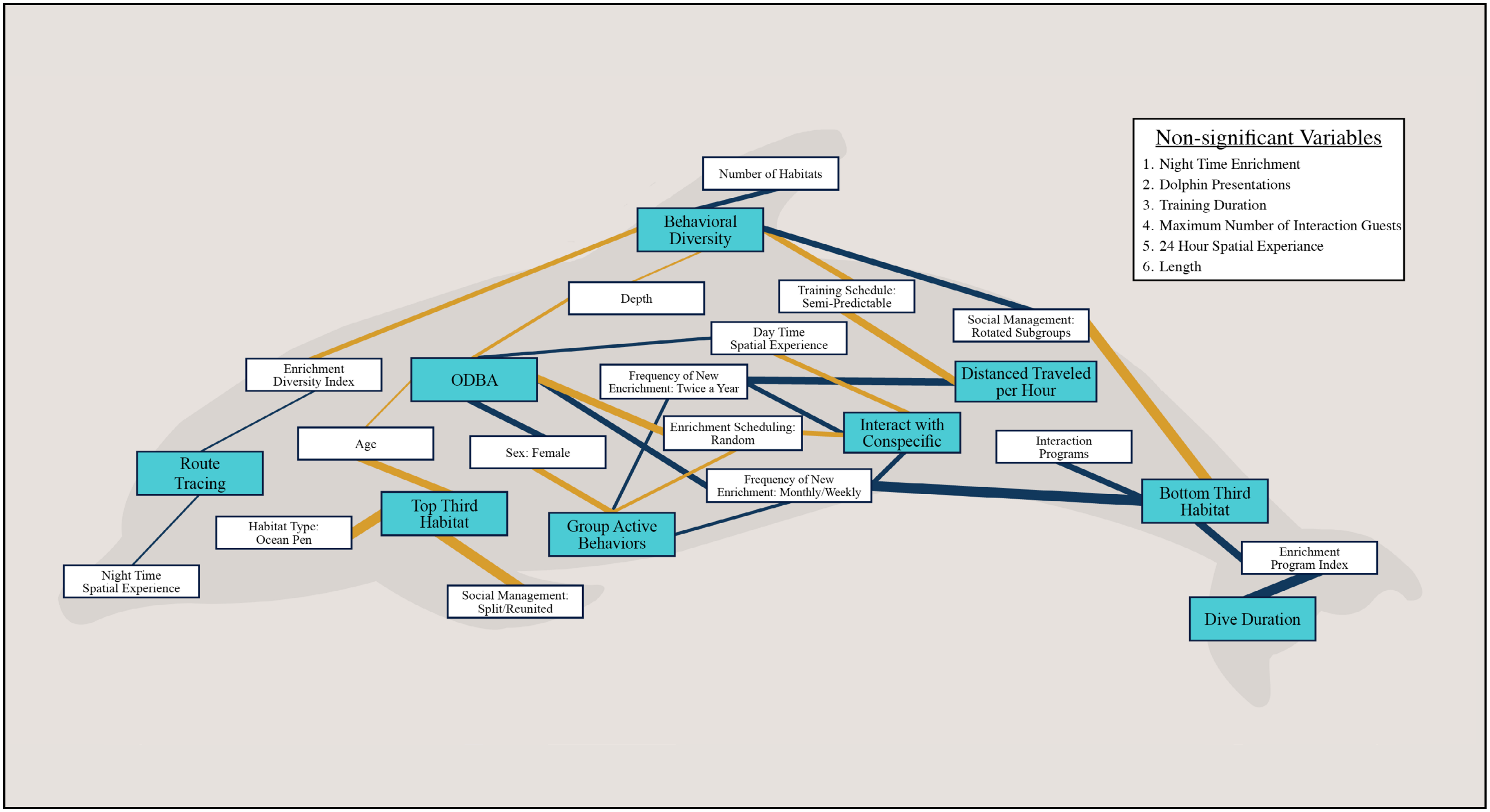

Lauderdale, Mellen, Walsh, Granger, & Miller (2021) Towards understanding the welfare of cetaceans in zoos and aquariums

Cetacean Welfare Study

Independent Variable Relationships

Click to see the significant variables.

All Variables | Demographic | Environmental Enrichment | Training | Habitat Characteristics

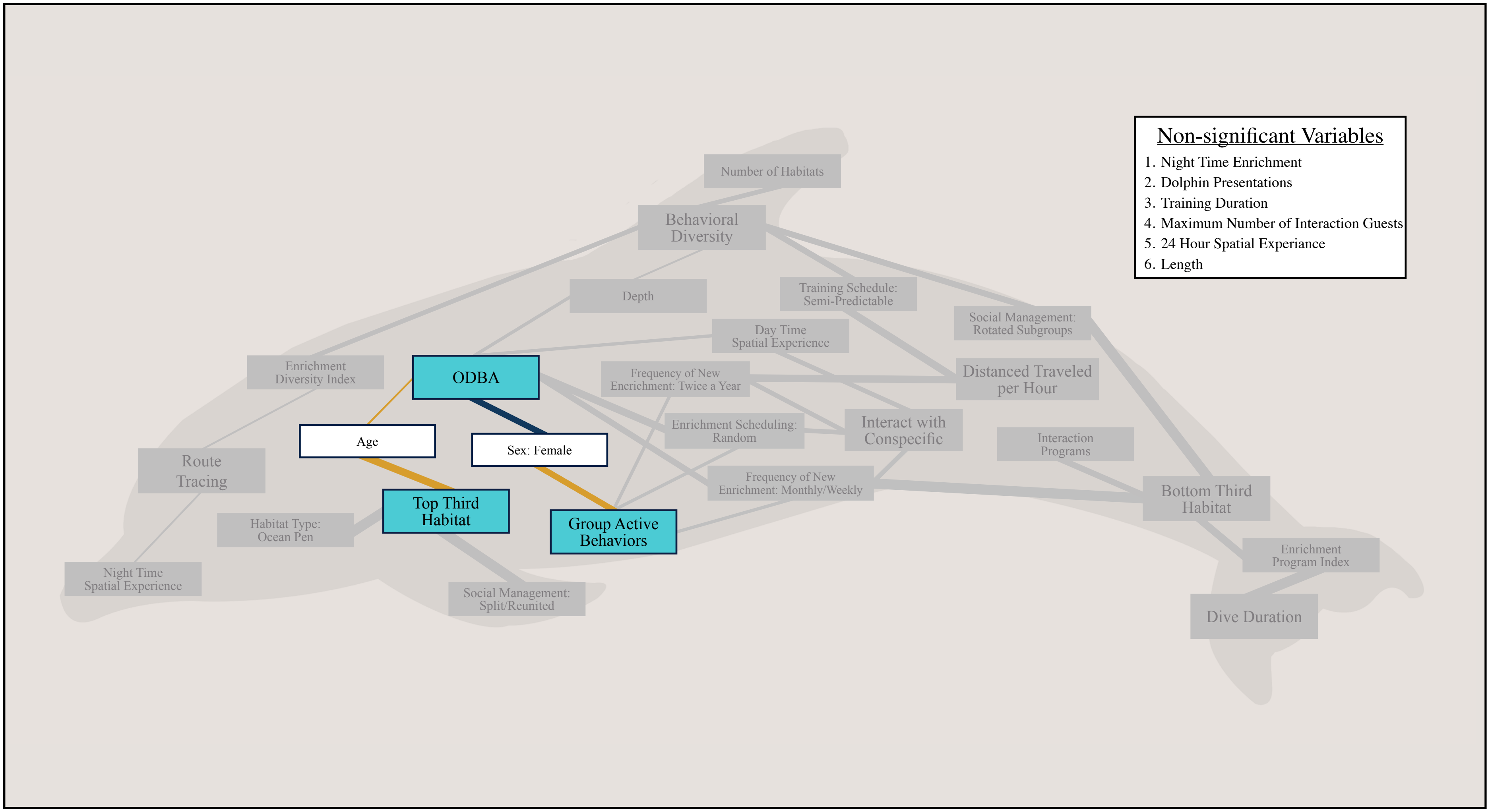

Lauderdale, Mellen, Walsh, Granger, & Miller (2021) Towards understanding the welfare of cetaceans in zoos and aquariums

Cetacean Welfare Study

Independent Variable Relationships

Click to see the significant variables.

All Variables | Demographic | Environmental Enrichment | Training | Habitat Characteristics

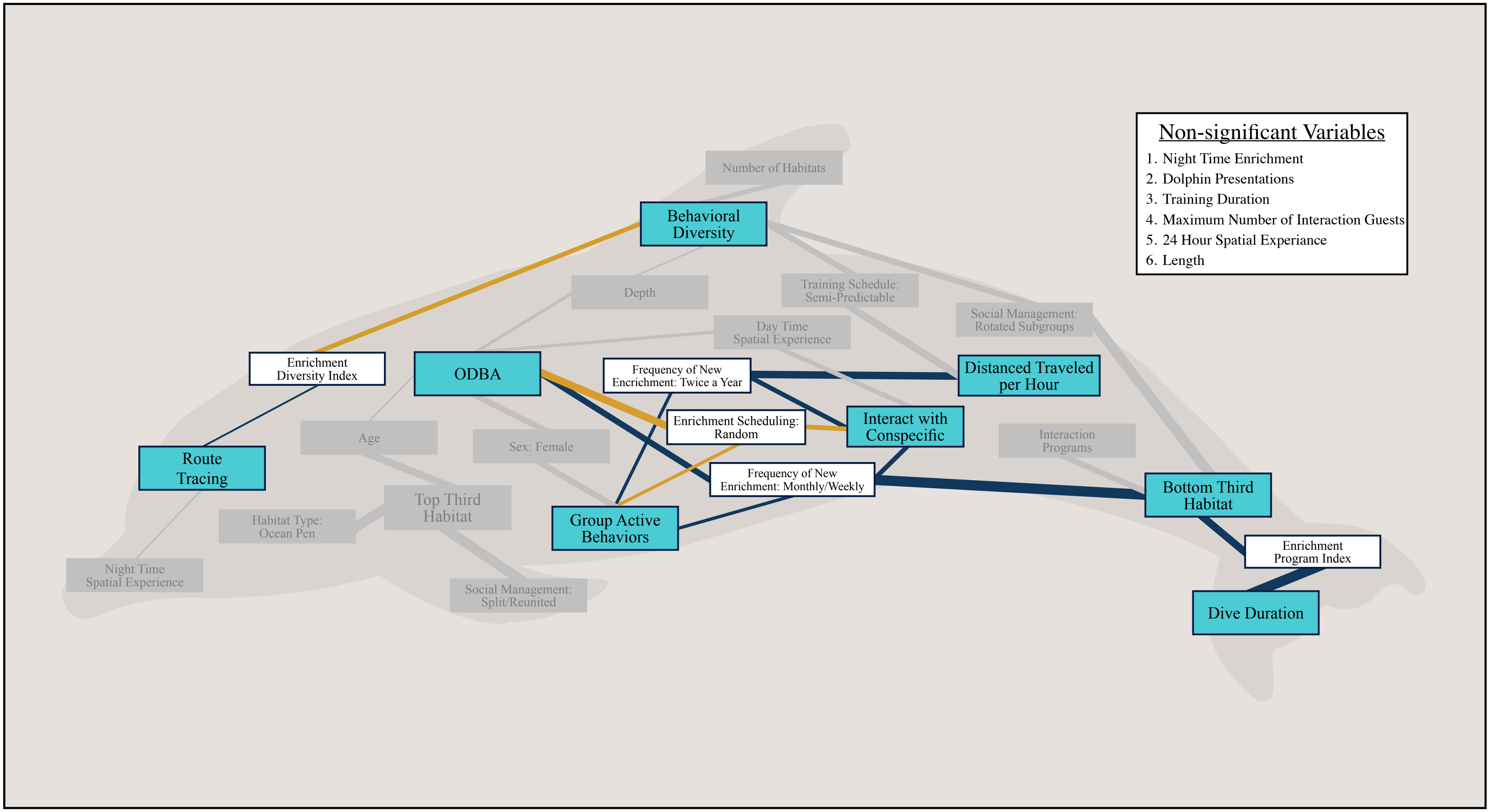

Lauderdale, Mellen, Walsh, Granger, & Miller (2021) Towards understanding the welfare of cetaceans in zoos and aquariums

Cetacean Welfare Study

Independent Variable Relationships

Click to see the significant variables.

All Variables | Demographic | Environmental Enrichment | Training | Habitat Characteristics

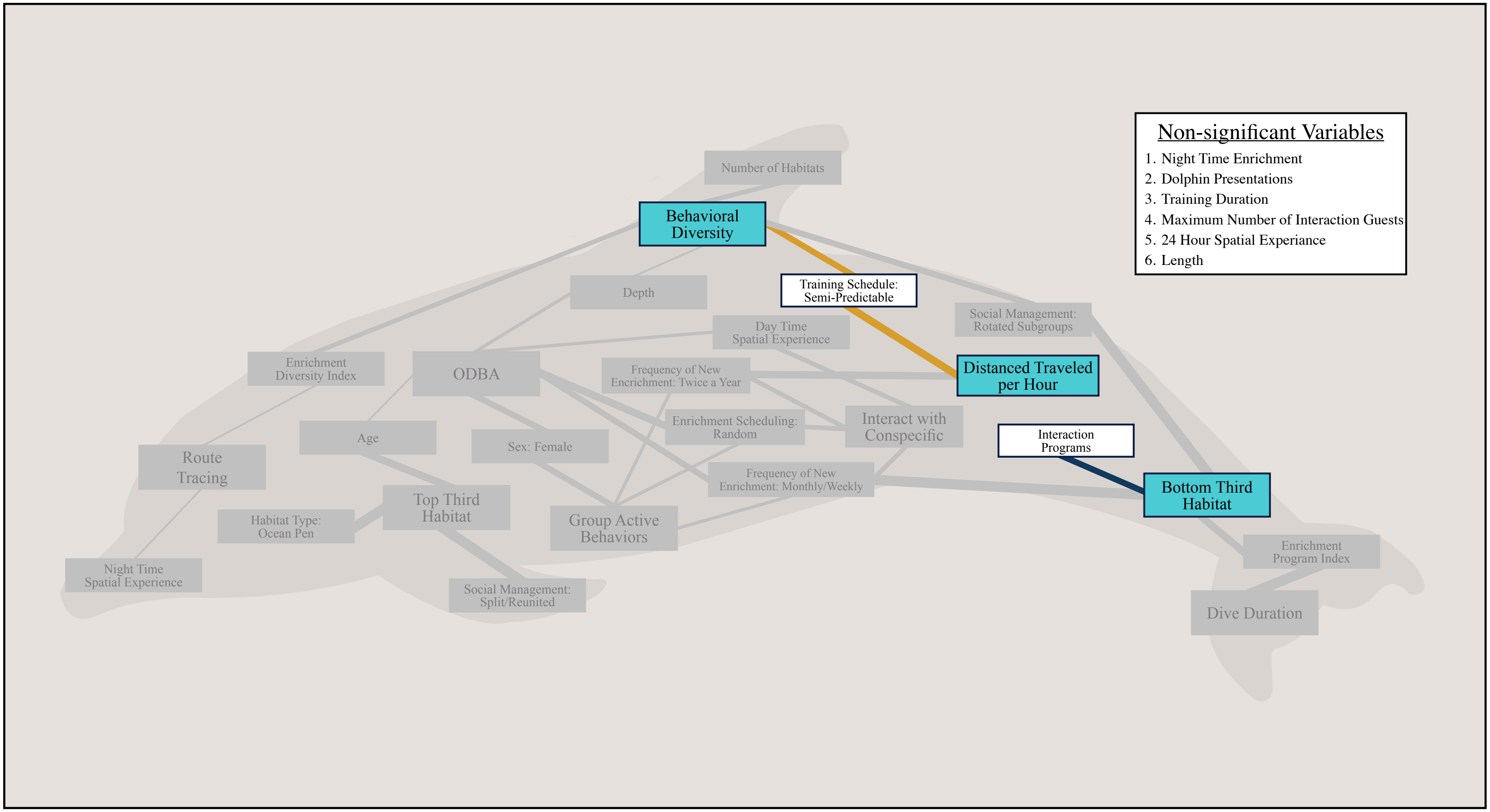

Lauderdale, Mellen, Walsh, Granger, & Miller (2021) Towards understanding the welfare of cetaceans in zoos and aquariums

Cetacean Welfare Study

Independent Variable Relationships

Click to see the significant variables.

All Variables | Demographic | Environmental Enrichment | Training | Habitat Characteristics

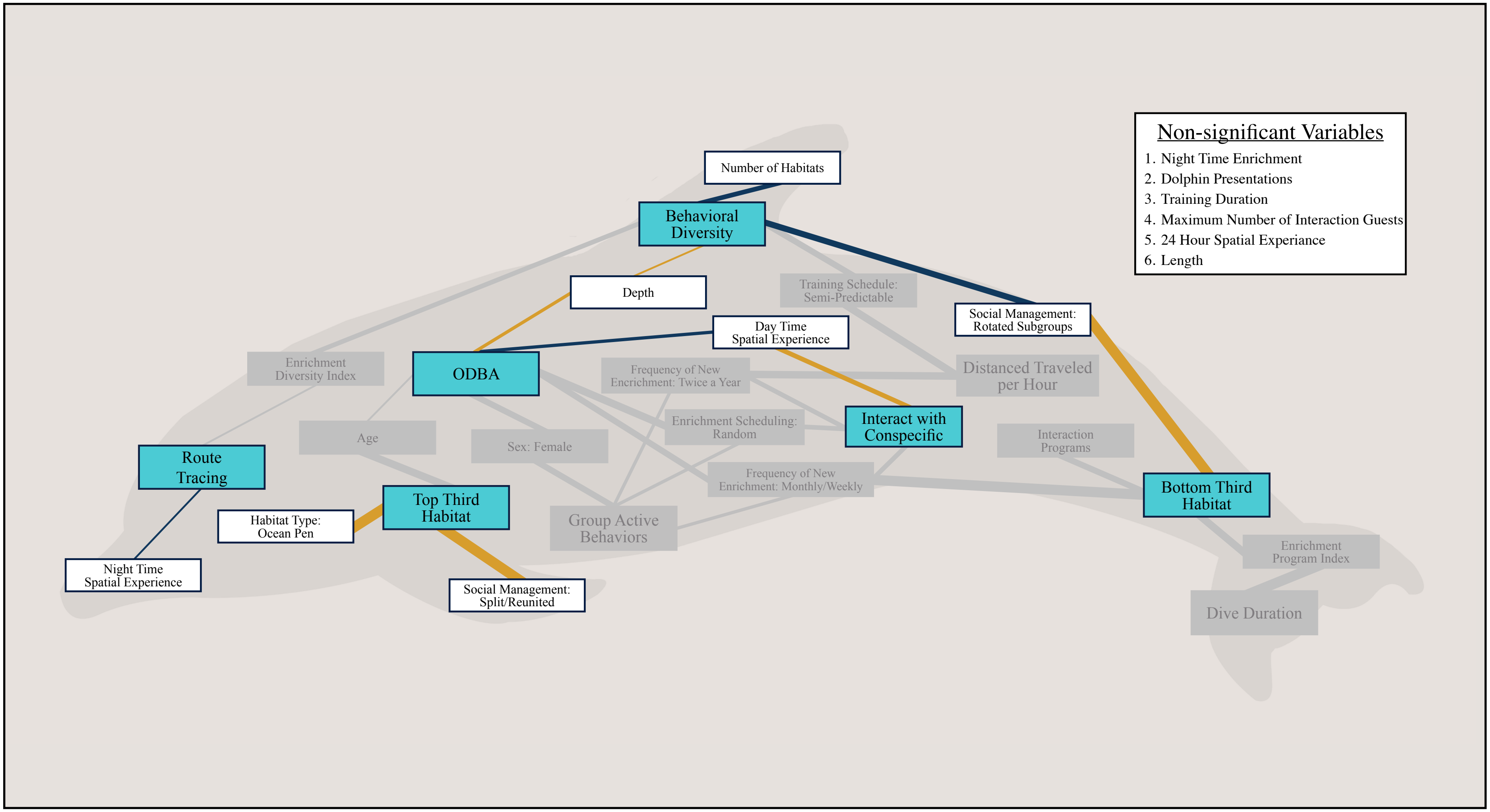

Lauderdale, Mellen, Walsh, Granger, & Miller (2021) Towards understanding the welfare of cetaceans in zoos and aquariums
