# Supplementary figures and images for: Towards understanding the welfare of cetaceans in accredited zoos and aquariums
Source: PLoS One. 2021 Aug 30;16(8):e0255506. doi: 10.1371/journal.pone.0255506 (PMC8404978; doi:10.1371/journal.pone.0255506)

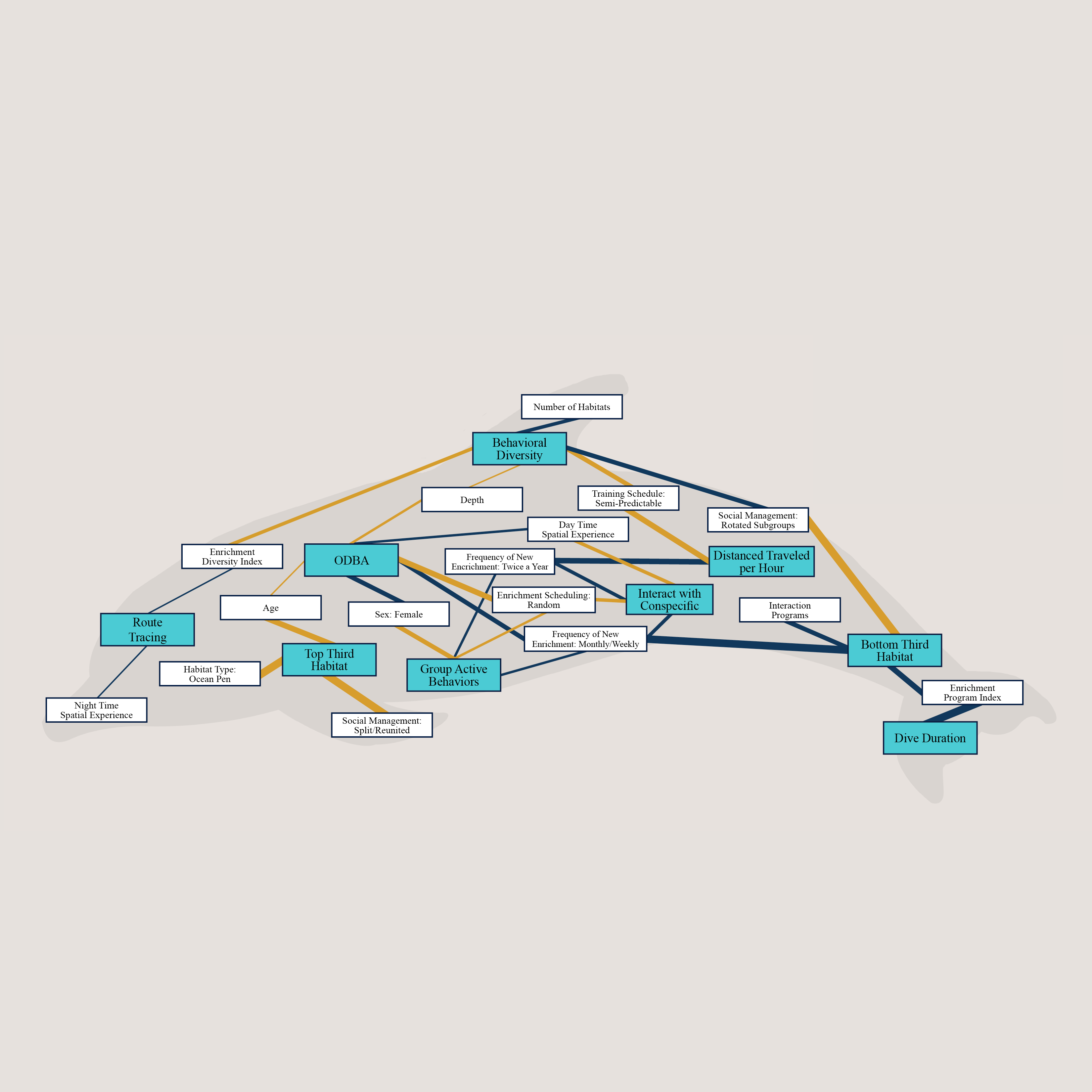

Supplement: S1 Fig — (TIFF) [file pone.0255506.s003.tiff]
